# Atropia in Collapse

**Published:** 1874-09

**Authors:** T. Curtis Smith

**Affiliations:** Middleport, Ohio


### ATROPIA IN COLLAPSE.

BY T. CURTIS SMITH, M.D., OF MIDDLEPORT, OHIO.

A little experience with this drug leads me to pen these few lines.

The full value of many of our old drugs seems not yet to be fully appreciated by all of the profession. We are learning something new every year of the action of such agents as ergot, digitalis, atropia, etc.

The action of belladonna or atropia in diseases of the eye has been known for many years. So also have many of its therapeutic applications in a general sense. But I am not aware that the use of atropia as a stimulant, or in collapse and conditions akin to it, is of more than recent date. Before first learning of its use in this way, I freely confess I was skeptical, but could still see that it was theoretically rational.

Atropia is a "spinal paralyzer; destroys excitability of the motor nerves; destroys muscular irritability; destroys the excitability of the sensory nerves; increases the action of the heart and the arterial tension by an excitation of the sympathetic; increases the action of the heart after division of the pneumogastric; increases respiratory movements." [Prof. R. Bartholow.]

In the foregoing quotation, we have the action of atropia on the animal economy, and in it we have actions that explain much of its seemingly contradictory effects. In destroying motor-nerve excitability, muscular irritability and sensory-nerve excitability, it rids the system of three elements that tend to the wasting of the animal tissues, and the rapid destruction of vital force, which, when not balanced by healthy repair, are liable to soon end in dissolution.

But we next notice that atropia increases the action of the heart, and increases arterial tension by an excitation of the sympathetic, and, moreover, increases the frequency of the respiratory movements, thus establishing a balance between respiration and the amount of blood circulating through the lungs. In addition to these effects, it has seemed to me to act as a powerful stimulant to the entire sympathetic system, and especially to the vaso-motor nerves governing the capillary circulation.

Without further speculations or statements of known physiological facts concerning its action, I will proceed to relate a few cases that have come under my observation in the last two years of experience.

In the early part of the summer of 1873, I was called to see a severe case of cholera morbus, that had been in progress some hours at the time of my arrival. The vomiting, purging and cramping was still in progress, and extremely severe. The patient was a middle-aged colored man, of good habits and good constitution. The vomiting and cramping soon ceased, under the use of morphia hypodermically, and the internal use of diffusible stimulants and alkalies, and I left the patient feeling much better, but greatly exhausted. The next day when I called he was suffering from a severe and profuse watery diarrhœa; no vomiting, but much tenesmus; pulse feeble, 120 per minute; skin feverish and dry; tongue furred, white, with very red base and edges; countenance pinched and sunken; patient unable to rise unaided. I gave stimulants and astringents freely, all of which were retained, but with no apparent benefit. Six hours later, I was hastily summoned, the messenger stating that the patient was dying. I found the patient with every symptom of collapse, which it is needless to name here, and the prospect seemed hopeless. Without any faith in the atropia, I gave hypodermically one-sixtieth of a grain. In a half hour from this dose there seemed to be an effort on the part of the system to rally. The dose was then repeated, and in a few minutes the cold, bluish, shriveled skin became warm and feverish; the face flushed; pupils dilated; mouth and throat dry, and the patient a little delirious. This latter symptom lasted only a little while. The patient rallied; had no more diarrhœa; slept quietly for two hours; took a little nourishment, and slept again. The diffusible stimulants were used freely, but were stopped after the first hour. After a few days of debility and weakness, the patient made a good recovery, without more diarrhœa.

This case strongly impressed my mind with the value of atropia in this condition of the system.

Case II.—July 6th, 1874, I was hastily called to see Mr. Mc., a man of stout build and good constitution and habits; a laborer, aged 28. He had been attacked about 1 A. M. with cholera morbus, which had evidently been severe. I did not see him till 9 A.M., eight hours after the attack. At that time his countenance was sunken, the face a livid, purplish color and cold, arms cold to the elbows, the lower extremities cold to the middle of the thighs, and the skin shriveled. The cramping had ceased measurably, but the vomiting was very persistent, and purging occasional and profuse, with little fœcal smell. I gave hypodermically, $\frac{1}{30}$ of a grain of atropia. In twenty minutes the extremities became warm, face flushed, vomiting and purging ceased and did not return, and reaction was well established. Nothing more was given, except a stimulant, which, however, was not used till the effects of the atropia begun to pass off. Recovery was rapid and without an untoward symptom, the patient being able to walk about the house the next day, in the evening.

Case III.—June 6th, 1874, I was called to see Mr. V., in consultation with Dr. J. B. Smith, of Syracuse, Ohio. Mr. V. had been sick three days with profuse rice-water vomiting and purging and cramping, for which he had been given the usual treatment, well adapted to such cases, but without any apparent benefit. The diagnosis was sporadic cholera. Whether correct or not, there was no symptom wanting to establish it as genuine, though there was not, and has not been, any other case of cholera in the vicinity for a year, and then but one or two. When we reached the house, we found him with a livid, sunken countenance; nose and ears cold; arms, feet and legs cold, shriveled and bluish, with little perceptible capillary circulation; respiration slow and sighing; pulse 60 per minute, and very weak; still vomiting and purging; rice-water emesis and stools, but not as frequently as formerly. None of the medicines, except a dose of calomel, seemed to be retained or prove beneficial. The stage of collapse was evidently at hand. After a few minutes' council, we gave him $\frac{1}{50}$ of a grain of atropia hypodermically. In thirty minutes there was a slight evidence of reaction, and we repeated the dose of atropia. In a few minutes after the second dose, reaction was fully established, so far as the circulation and warmth was concerned; but there was still great exhaustion. Diffusible stimulants were now given freely. Under the atropia, and before any other agent was given, the extremities became warm, and lost their shriveled, bluish appearance; the face became flushed, and the head ached severely; the pupils in this case did not dilate, but mouth and throat became dry. There was no more vomiting, purging or cramping, and from this time the patient made a steady recovery.

CASE IV.—This was a case of genuine cholera, not my patient, though I saw him with others while in a stage of collapse, or rather as it was approaching. Atropia was administered, but how much I do not know. The case terminated fatally. I doubt if any remedy could have reached this case, as it was very severe, and under very poor circumstances for proper treatment.

The foregoing are a part of the cases in which I have used or seen used the atropia in collapse, and my limited experience has led me to place confidence in the agent in this and similar conditions.

While I have great confidence in this agent for this purpose, I do not wish to convey the idea that it will always overcome collapse, even where it is not the result of organic lesions, but simply that I deem it our most powerful means of combatting that unhappy condition. Neither should it be used to the exclusion of such adjuvant measures as are calculated to restore reaction, though I have seen it prove a perfect success where everything else was left off.

Apropos to what I have written is the following quotation, taken from *The Clinic*, August 2, 1873, p. 57, taken from the

*American Practitioner*, July, 1873, by "Dr. R. Sanders, of Padukah, Kentucky, a practitioner of age and very great experience." He says: "In the recent outbreak of cholera in Padukah, I treated a number of cases by sulphate of atropia hypodermically—one-fiftieth to one-thirtieth of a grain in water—with the happiest results. The more distressing symptoms—vomiting, purging and cramps—were relieved almost at once, followed by refreshing sleep, continuing in some cases for several hours. I found these, however, to follow only when the atropia was used in sufficient quantities to produce the specific scarlatinal rash, dry throat, and dilatation of the pupils. In some cases, the relief afforded was astonishing; the skin grew warm; the pulse rose; the surface, previously clammy and shriveled, assumed its natural condition, and in some instances the patient slept soundly for ten or twelve hours, the bowels remaining undisturbed during the entire time. Of course, you will not understand me as advocating the exhibition of atropia to the exclusion of all other means, especially the use of calomel, to which I attach much importance."

On this authority I first made use of atropia in this class of cases, and can speak very commendably of its use so far.

